# 

**Published:** 1866-07

**Authors:** 


### CONTEN S FOR JULY.

ORIGINAL CONTRIBUTIONS.

On Progressive Locomotor Ataxy. By Benjamin Durham, jr., M.D...p. 289
A Case of Progressive Locomotor Ataxy. By J. P. Ross, M.D.......... 294
Report of the Committee on Diseases of the Eye. By E. L. Holmes, M. D.......... 296

PROCEEDINGS OF MEDICAL SOCIETIES.

American Medical Association.......... 307
Iowa State Medical Society.......... 317
Illinois State Medical Society.......... 324

EDITORIAL.

Book Notices.......... 332

